# Barriers and facilitators to implementing dementia care mapping in care homes: results from the DCM™ EPIC trial process evaluation

**DOI:** 10.1186/s12877-019-1045-y

**Published:** 2019-02-08

**Authors:** Alys W. Griffiths, Rachael Kelley, Lucy Garrod, Devon Perfect, Olivia Robinson, Emily Shoesmith, Joanne McDermid, Natasha Burnley, Claire A. Surr

**Affiliations:** 1 Centre for Dementia Research, School of Health and Community Studies, Leeds Beckett University, Leeds, LS1 3HE UK; 2 Oxford Health NHS Foundation Trust, Oxford, UK; 3 Wolfson Centre for Aged Related Diseases, King’s College London, London, UK

**Keywords:** Person-centred care, Training implementation, Psychosocial intervention, Dementia care mapping, Care homes, Process evaluation

## Abstract

**Background:**

Psychosocial person-centred interventions are considered best practice for addressing complex behaviours and care needs such as agitation and anxiety, and for improving the quality of life of people with dementia in care homes. Dementia Care Mapping (DCM™) is an established practice development tool and process aimed to help care home staff deliver more person-centred care. To date, few studies have evaluated the efficacy of DCM™ and have found mixed results. These results are suggested to be the outcome of intervention implementation, which may be impacted by a range of factors. This study reports the barriers and facilitators to DCM™ implementation in care homes found during the process evaluation conducted as part of a randomized controlled trial.

**Methods:**

Eighteen of the 31 DCM™ intervention care homes were recruited to participate in the embedded process evaluation. Semi-structured interviews were conducted with 83 participants, comprising care home managers, trained DCM™ users (mappers), expert external mappers, staff members, relatives, and residents.

**Results:**

Barriers and facilitators to DCM™ implementation were found at the mapper level (e.g. motivation and confidence), the DCM™ intervention level (e.g. understanding of DCM™) and the care home level (e.g. staffing issues, manager support). Further barriers caused by the burden of trial participation were also identified (e.g. additional paperwork).

**Conclusions:**

Implementing DCM™ is complex and a greater consideration of potential barriers and facilitators in planning future studies and in practice could help improve implementation.

**Trial registration:**

Current Controlled Trials ISRCTN82288852, registered 16/01/2014.

## Background

Successfully implementing complex psychosocial interventions in care homes is known to be challenging, as such interventions often involve multiple components [1] and may require staff to lead or support implementation. Process evaluations, which aim to understand whether interventions have been implemented as intended, are recommended for multisite randomized controlled trials (RCT), where between-site differences may exist in intervention delivery and receipt [2]. Therefore, understanding the intervention process and implementation issues is important in considering research study design and implementation. Furthermore, this can provide an insight into study results, particularly within large-scale clinical trials.

Dementia Care Mapping (DCM™) is an observational tool set within a practice development process, which aims to support staff working in formal care settings to record and understand the care experience of people with dementia and to use this as a basis for person-centred care planning. However, despite its widespread use in care home settings [3], there is very little robust evidence published about factors that contribute to its successful or unsuccessful implementation [4].

To date, there have been six published studies that have examined the efficacy of DCM™ for improving the delivery of person-centred care for people living with dementia in care homes. Two cluster RCTs have found beneficial effects of DCM™ on agitation and falls [5] and neuropsychiatric symptoms including agitation and psychosis [6]. However, a more recent RCT found no benefit of DCM™ over care as usual for agitation amongst people living with dementia, although benefits to the quality of interactions between residents and staff were found [7]. The remaining three studies were less methodologically rigorous. Two small pilot studies found a reduction in anxiety and verbal agitation [8] and depression and agitation [9] for care home residents living with dementia following use of DCM™. A quasi-experimental study with three arms (DCM™ implemented for 2 years prior to commencement of the trial, DCM™ as a trial introduced intervention, and quality of life care planning control), found no benefits of the DCM™ arms over control for quality of life or reduction of behaviours that care staff may find challenging [10].

Of these six studies, only two have included a process evaluation [7, 11], to allow the understanding of DCM implementation fidelity and processes. One of these in particular, highlighted issues with intervention fidelity within some of the clusters and recommended that process evaluations be conducted in further RCTs, in order to assess whether the intervention has actually been delivered as intended [7]. This is particularly pertinent in DCM™ research, where the six studies have been conducted within five different countries, each implementing DCM™ in a unique way, making it difficult to draw comparisons between the findings. Furthermore, studies using explanatory ‘ideal condition’ designs, where researchers led the implementation of DCM™ (e.g. [5]) were more likely to show benefits for people living with dementia than studies using the standard DCM™ model of care home staff led implementation (e.g. [7]), a finding which process evaluations could help to explore.

A recent systematic review of DCM™ implementation in care homes found a scarcity of research in this area and reported that implementation challenges were reported in the few published studies discussing process issues [12]. This review highlighted some common factors across studies that were thought to improve the delivery of DCM™ including selection of individuals to implement DCM™ (known as mappers), having appropriate preparation and support during implementation, and effective leadership within the organisation to deliver person-centred care. Common challenges were also highlighted, including time required to implement DCM™, staff team resistance to change, and lack of managerial or organisational support [12].

The present paper reports on the process evaluation of the EPIC pragmatic, cluster RCT, conducted in fifty care homes in the United Kingdom [4]. The trial design was based on the commonly used staff-led model of DCM™ implementation in UK care homes, whereby two staff members trained as part of the trial lead implementation, as opposed to researchers. The integral process evaluation followed the Medical Research Council guidelines [13] and aimed to understand implementation dose, fidelity and process issues across sites. This paper reports on the perceived barriers and facilitators to DCM™ implementation.

## Method

### Design

The design of the EPIC trial is reported elsewhere [4]. To provide a summary of the trial, 726 residents from fifty residential, nursing and dementia care homes across three areas of England who provided care for people with dementia, were recruited, with a further 216 residents recruited from these care homes at 16-months follow up due to higher loss to follow up than anticipated. Care homes were randomised on a ratio of 3:2 to intervention or control (see Table 1). Randomisation was conducted immediately following baseline data collection, using an automated randomisation system. This operated through a computer-generated programme, which ensured arms were balanced for the following care home characteristics; home type (residential/nursing/specialist dementia), home size (≥40 bedrooms/< 40 bedrooms), delivery of dementia awareness training by the research team (yes/no) and recruitment area (West Yorkshire/Oxford/London). Intervention homes were asked to complete DCM™ alongside usual care and control homes were asked to continue with usual care. in line with standard DCM™ practice, two staff members from each intervention care home were trained to use DCM™ and then asked to implement three DCM™ cycles, each comprising of briefing; observation; data analysis, reporting and feedback; and action planning. These cycles were scheduled at 3-months (or as soon as practicable), 8-months and 13-months post-randomisation. During use of DCM™^,^ mappers were expected not to be involved in the delivery of care. In agreeing to participate in the trial, care home managers approved that staff would be paid for all shifts where they undertook DCM™. Mappers were asked to observe for as long as they were able on a single day or over a week, up to a maximum of 6-h. They were asked to observe between one and five residents, depending on their confidence in using the tool. The first cycle was supported by an external DCM™ expert mapper, who attended the care home to provide practical support for the briefing, observation, feedback and action planning and provided additional support for data analysis and report writing remotely. This was to support standardised intervention implementation across all care homes. To support intervention fidelity and its measurement, care homes were provided with guidelines which included standardised templates for recording attendance at briefing and feedback sessions and for DCM™ reporting and action planning. Additional mechanisms for supporting intervention adherence included sending SMS reminders and paperwork to mappers ahead of each cycle, and provision of telephone support from the DCM™ intervention lead.

**Table 1:** Care home and resident demographics

	Intervention	Control	Total
Care home type			
Residential/nursing	20 (64.5%)	11 (57.9%)	31 (62%)
Specialist dementia care	11 (35.5%)	8 (42.1%)	19 (38%)
Number of permanent residents M(SD)	32.9 (14.02)	30 (11.27)	31.8 (12.98)
Percentage of residents with dementia M(SD)	74.2 (22.48)	83.1 (21.21)	77.7 (22.21)
Average resident:staff ratio daytime Med(Range)	4.7 (2.5, 10.5)	5.2 (3.0, 8.8)	4.8 (2.5, 10.5)
Average resident:staff ratio night time Med(Range)	9.7 (2.9, 15.3)	9.5 (3.3, 17.5)	9.7 (2.9, 17.5)

The mappers recruited in each care home were selected by the Manager, following discussion with the research team. All mappers were required to be permanent members of staff and judged by the Manager to meet the criteria for being a mapper (e.g. English language skills, confidence in delivering briefing and feedback sessions, ability to use data analysis package). Mappers received standardised 4 day training in DCM™ and did not receive any financial incentive for this.

Withdrawal of one or both of the mappers occurred in 17 homes (55%). At 16-month follow-up 14 homes (45%) had two trained mappers still in post, 7 had one mapper (23%) and 10 (32%) had no mappers. While there was funding to train additional mappers this only occurred in one home due to insufficient time before the end of the trial to train further mappers, being unable to identify a suitable replacement mapper or the consented mapper being unable to attend scheduled DCM™ training due to personal or organisational reasons.

Semi-structured interviews were conducted with residents, the care home Manager, DCM™ mappers, staff, and relatives in 18 of the 31 intervention homes.

We selected 18 homes to provide a broad and manageable sample size. Purposive sampling was used to select care homes with a range of characteristics that may have affected DCM implementation (e.g. variations in size and type of care home) and to select homes that had implemented different doses of DCM™ (0–3 cycles), in order to explore the factors associated with implementation in greater detail. Interviews took place after all completion of all outcome data collection in each care home.

### Participants

Interviews were conducted with a range of staff including care home managers, mappers, staff members, residents and relatives, as well as the expert mappers who had supported staff during their first DCM™ cycle. A total of 83 participants were interviewed; 17 care home Managers, 25 DCM™ mappers, 27 staff members who held a range of roles and had varying degrees of involvement with the intervention, 6 relatives, 2 residents and 6 expert mappers (see Table 2 for distribution of participants). The length of interviews varied from 3 to 38 min, dependent on the interviewee’s knowledge and awareness of the intervention.

**Table 2:** Distribution of participants across care homes

Care Home	Managers	Mappers	Staff Members	Relatives	Residents
CH1	1	1	3	1	
CH2	1	2	1		
CH3	1	2	1	1	
CH4	1	2	1	1	
CH5	1	2	2		
CH6	1	1			
CH7	1	2	1		
CH8	1			1	
CH9		1	4	2	1
CH10	1	2	1		
CH11	2	1	2		
CH12	1	2			
CH13	1		2		
CH14	1	1			
CH15	1	2	1		
CH16	1	1	4		1
CH17		1			
CH18	1	2	4		

Identification of staff to approach was undertaken by the researchers in conjunction with the care home Manager. This included identification of staff members who had played a key role in intervention delivery. Staff members were then provided with information about the study before deciding whether to participate or not. Mappers who had no longer worked in the care home were not interviewed.

Residents who had capacity to give informed written consent to participate in an interview were invited to participate by a staff member and researcher. Due to the high attrition rate and few residents being assessed as able to give informed consent, two residents agreed to participate in an interview.

Relatives or friends who had regularly visited their relative (at least once per month during the trial), were invited to participate. Relatives or friends of residents who had died during the trial were not contacted.

### Data collection

Semi-structured interviews were conducted within each care home, with telephone interviews offered to relatives and friends. Resident interviews were brief and used a conversational style. All other interviews were informed by a topic guide designed by the research team in conjunction with the public and patient involvement group (see Table 3 for a summary). The majority of interviews were conducted with a single individual, but some were completed in pairs or small groups, based on participant preference. Interviews focused on the experiences of DCM™, with participants encouraged to discuss the various stages of implementation; specifically, what barriers and facilitators they faced at each stage and the impacts of implementation.

**Table 3:**
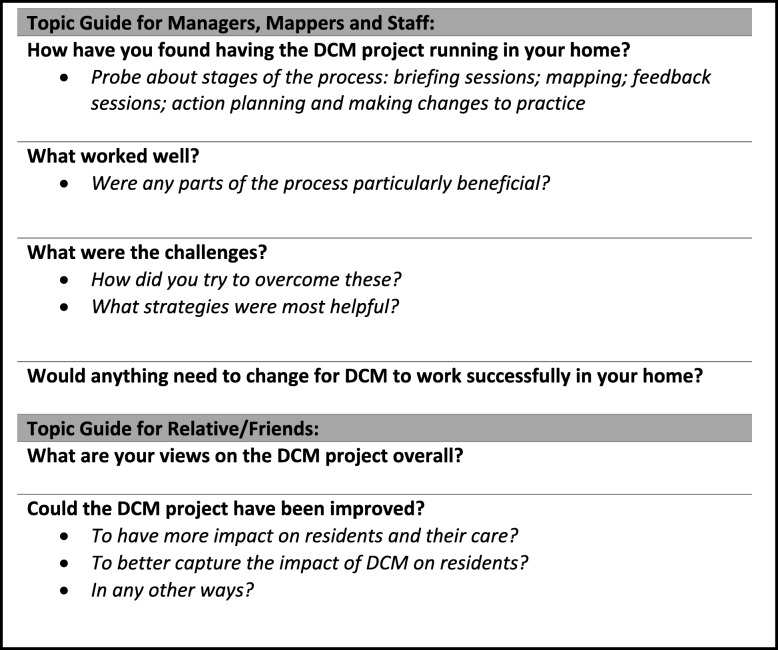
Completion of intervention components by cycle by furthest cycle component reported as completed (*n* = 31)

Interviews were audio recorded and transcribed by a researcher independent to the study. Any potentially identifying information about participants or care homes was removed during transcription.

### Data analysis

Data analysis followed a Framework Analysis approach [14]. The research team developed a coding matrix, which guided and created a structure for further data analysis. The focus of the coding matrix was on experiences of utilising and implementing DCM™, specifically, identifying patterns and variations in, as well as barriers and facilitators to implementation. This paper reports on the data around barriers and facilitators to implementation.

Each transcript was independently coded and analysed by two members of the research team – one from the research hub who had completed trial data collection in the care home and one who had not visited the care home. The researchers discussed their analysis and came to agreement on where quotes should be placed within the framework. The development of the coding categories continued throughout data analysis, informed by the emerging themes and analytic thoughts of the researchers. Codes and themes were compared and contrasted across care homes and between different types of participants, to develop an in-depth and contextualised understanding of the barriers and facilitators to the implementation of DCM™.

## Results

As a first step, DCM™ implementation was examined by the research team. DCM™ implementation was poorer than expected, even with DCM™ expert mapper support. Sixteen (51.6%) of care homes completed only one cycle to an acceptable level, 4 (12.9%) completed two cycles to an acceptable level and 4 (12.9%) completed all three cycles to an acceptable level. Seven care homes (22.6%) did not complete a full intervention cycle, with three (9.7%) of these not completing any of the intervention components (see Table 3 for a summary of DCM™ implementation).

The findings demonstrated that implementing DCM™ in care homes is complex, and there are many factors that may facilitate or prevent successful implementation. Managers, mappers, expert mappers and staff members identified barriers and facilitators; no residents or relatives highlighted any barriers or facilitators.

Barriers and facilitators were identified at four levels; (1) care home, (2) mappers, (3) intervention, and (4) trial (see Table 4 for a summary of sub-themes within these themes).

**Table 4:** Identified themes and sub-themes that emerged as facilitators and barriers

Theme	Sub-Theme
Care Home	Staffing related issues Time and competing priorities Care home manager Staff motivation, engagement and openness to change Mapper status and leadership skills
Mappers	Choice of mappers Mappers experiences
Intervention	Understanding of DCM™ Complex nature of DCM™
Trial	Expectations of the trial Input from DCM™ expert mappers

### Care home barriers and facilitators

Care home contextual factors influenced the degree to which DCM™ was successfully implemented within each care home. This included broad issues, such as the type of setting and staffing levels, and more specific issues such as the availability of computers in the home and funds to support implementation.

#### Staffing related issues

Managers of residential homes reported being at a disadvantage due to their lack of nursing qualified staff members, who might hold expertise to help facilitate implementation. This suggests implementation may perceived as being easier in larger nursing or dementia-specific care homes with greater numbers of staff who are qualified nurses or more experienced in dementia care.

*“Because we are only a residential home, erm, y’know, we haven’t got nurses and stuff so my staff aren’t that confident anyway… I’m glad we got involved because we got a lot out of it, I’m just disappointed that we weren’t able to continue.” (Manager)*

However, one expert mapper highlighted the lack of consistency in staff member’s perceptions of care home settings where DCM would be more easily implemented.

*“I think it was often by, by trained nurses saying this would work in a care home, not a nursing home. That was my experience. I have heard that in other situations about other tools. That you know, this will work for people not so disabled, or more disabled.” (DCM™ expert)*

Management of smaller homes suggested that they struggled to accommodate the cover necessary to facilitate DCM™, whereas larger care homes that were well staffed were able to build the time into their rota.

*“Because we are only a small home we have only got a small amount of staff, trying to have staff to be supernumerary for all that length of time, err, was difficult.” (Manager)*

Across all care home settings, low staffing levels and high staff turnover were reported as a barrier to the implementation of DCM™. The consistency of staff involvement is necessary to understand the changes over time for residents and also to implement changes as a result of DCM™ in a consistent manner.

*“Care homes are really, really busy. Turnover of staff in care homes can be quite dramatic at times, and the realities are there’s other pressures on them isn’t there.” (DCM™ expert)*

Of particular importance was the turnover of trained mappers, which led to delays in implementing DCM™. This also happened during or after the first DCM™ cycle, which was particularly challenging since in all but one of the homes where this occurred, a suitable replacement mapper was not able to be identified and trained.

Additionally, the attrition of mappers had an impact on the confidence of remaining mappers, leaving some feeling overwhelmed by what was required of them. DCM was often considered to be a complex and time-consuming process due to the paperwork, length of training course and individual elements of the mapping process. These requirements of implementing DCM as a lone mapper were considered overwhelming alongside their normal care home duties, whereas having the support from a second mapper eased the process.

*“It were definitely better having two rather than having just doing it on my own, because I think I would’ve struggled a lot more” (Mapper)*

Withdrawal of one or both of the mappers occurred in 17 homes (55%). The reasons for withdrawal were resignation from the care home, ill-health/long-term sickness, maternity leave, and in one home, both mappers withdrew due to lack of management support to map.

*“The other girl I was supposed to be doing it with, wasn’t available most of the time through my first one, and then she left! So, I’ve been on my own more or less through the whole three sessions.” (Mapper)*

#### Time and competing priorities

Time constraints and competing demands often overshadowed the motivation of mappers in completing cycles of DCM™. For example, mappers suggested that whilst they were able to complete the mapping hours, they often struggled to find the time to finish the cycles particularly the report writing and feedback sessions.

*“It’s all getting the time and people are rushed off their feet in the morning and they haven’t got time to come here for half an hour.” (Mapper)*

Managers referred to adaptations required to make DCM™ fit in their home. This included suggested or actual adaptations to the process of DCM™ itself, such as shorter observations, and hypothetical or actual adaptations to staff workloads, such as changes to rotas.

Managers’ reported that external demands placed upon the home during the study period, including regulatory reports and issues with staffing had to take precedent over DCM™ implementation.

*“We get inspected by health and safety, infection control, the social workers, CQC [regulatory authority] come, social services come, y’know, it’s just ongoing and they are all asking for more paperwork… we are struggling to do the paperwork that we have got already.” (Manager)*

Whilst nursing homes noted additional complications due to the demands of the setting, the value of DCM™ was acknowledged.

*“I think it’s just the work load, really. The amount of work there is sometimes, and with it being a nursing home - the intensity of the workload. Obviously, we have a lot of very poorly people sometimes.” (Mapper)*

#### Care home manager

A key individual in successful implementation of DCM™ was the care home manager. Whilst managers were not always directly involved in implementation, they generally had responsibility for rotas, allocation of staff workload and supervision of the mappers. Therefore, successful engagement of the management team was key to effective implementation of DCM™. Conversely, where this was not achieved, it created barriers for the mappers.

*“I think management support, you know, it can either be amazing when it’s amazing or it can be a real difficulty if the manger isn’t supportive.” (DCM™ expert)*

Generally, interviewees perceived a lack of support from managers.

*“as far as I’m aware they were just pushed to be doing other stuff and it kept getting left and left and not done..” (Staff Member)*

Managers needed to be willing to support implementation and have an awareness of the time required by mappers for this process.

The hierarchical nature of care homes sometimes acted as a barrier in the process, meaning that mappers were unwilling or felt unable to challenge the manager. This was particularly pronounced where there were difficulties in the relationship between managers and mappers.

*“It’s mainly from a confidence perspective, [they] were clearly not confident to challenge a manager who was not supporting.” (DCM™ expert)*

Conversely, where managers were engaged with DCM™, this facilitated the process and helped mappers to make changes based on what was observed during the cycles. Furthermore, where managers valued DCM™, they could see clear benefits from its implementation. For example, one manager believed that it was a key tool to help with regulatory ratings of care home quality.

*“They were very clear that they thought DCM™ was fantastic, because they saw it as a way of improving the quality of their care to take their home CQC [UK regulator of care homes] rating from good to outstanding.” (DCM™ expert)*

#### Staff motivation, engagement and openness to change

Motivation and enthusiasm were crucial in the successful implementation of DCM™. Expert mappers emphasised that when managers and staff teams were motivated to be involved in the DCM™ process, mappers were more likely to implement DCM™.

*“The manager would come in and you know be really enthusiastic. They came to the briefing, everybody was at the briefing, the whole home, the manager of the home, do you know what I mean. The company really bought, really bought in to DCM™. And the two girls, the two mappers were just really enthusiastic about it, … and really, really tried their hardest.” (DCM™ Expert)*

It was beneficial to undertake the first cycle of DCM™ soon after training was completed, perhaps due to capturing the initial motivation and confidence of the mappers.

*“They went for that training … then there was a gap and I kind of think if they had just gone straight in and done the mapping, they might have done it. But I feel that when a few weeks passed, they were struggling to say how we do this… maybe they didn’t have the confidence, you know what, to roll it out.” (Manager)*

As DCM™ was delivered within care homes, effective implementation was influenced by the level of engagement from care home staff. It was beneficial if staff were open to feedback from observations, and were cooperative in formulating new action plans. Some mappers had the ability to encourage multiple staff members to attend and engage in feedback sessions. Those who held a senior role may have found this easier, due to their reputation within the home.

The importance of a ‘whole home’ approach, involving staff members from a range of disciplines attending feedback sessions, was highlighted.

*“There was a really big crowd actually, and it did include lots of different disciplines of staff, including the painter and decorator and maintenance man, which was great.” (DCM™ expert)*

As the intervention was operating at a care home level, high levels of staff engagement was required in DCM™ feedback and agreeing action plans in order to initiate change.

*“You really have to get quite a few people across the organisation thinking in the same way to sort of drive that change.” (Manager)*

Staff engagement was attained through multiple strategies. These included ensuring staff understood DCM™, it’s objectives and the output of the mapping, giving feedback during staff meetings to maximise staff involvement, providing staff members with positive as well as negative feedback to ensure that staff remained engaged, and demonstration of the benefits of DCM™ within the care home.

*“We sort of ended up picking two or three very small examples of people who were very happy or very sad and just focusing on those, describing in laymen’s terms... They did take it in a positive way because they’d been, initially we said it’s for all our residents’ well-being.” (Mapper)*

Some staff members held a negative attitude towards the DCM™ process which acted as a barrier to ‘whole home’ engagement. If DCM™ was not perceived to be a priority, staff often did not take time to learn about and understand the process, which made it particularly difficult for mappers to try and implement any changes.

*“I felt that the ways that people had been working prior to that, the culture of the place, whilst there was a lot about it which I would really commend it for, there were definitely some things that needed to be looked at. And I felt that there was a reluctance to look at that. And there was quite a lot of defensive response.” (DCM™ expert)*

The validity of DCM™ was questioned by some staff members, particularly when residents were often unwell or they considered that DCM™ did not suit the residents they provided care for.

*“…some of our residents are quite, quite poorly so it doesn’t work for them, it just depends how well they are.” (Staff member)*

Collective reflection on DCM™ feedback sometimes made staff feel a part of the process and helped to break down potential barriers and mistrust. For example, with the process of being observed by inspectors and receiving feedback, which staff and the care home generally, could have past negative experiences of, DCM™ helped to change these perspectives.

*“In most cases when it happens, it’s a negative experience because there’s inspectors from various organisations, so I think it wasn’t until we started giving feedback and there was quite a bit of positives in there that the staff really got engaged with the process.” (Manager)*

#### Mapper status and leadership skills

The respect held for the mappers within the home was important for staff engagement. The peer led method of the feedback sessions facilitated staff engagement in the DCM™ process. Managers noted that this effect was observed where the mapper was respected within the home.

*“It’s people that you know and peer-led, it’s, you know, it’s not like somebody from outside coming and talking with them, it engages the staff.” (Manager)*

When mappers found it difficult to engage the staff team, change was difficult to implement. In one care home, following completion of the first components of the cycle, the mappers were unable to engage with staff members to progress further.

*“The second time around we held a meeting and nobody came … We did try like you know individual, a few minutes at a time, but I don’t think they took it seriously enough, do you know what I mean?” (Mapper)*

Further difficulties in implementing DCM™ arose when staff did not support the mapper. In one home, there was a divide between staff members, with a proportion supporting the mapper and a proportion not supporting the mapper. Subsequently, this caused challenging feedback sessions, resulting in some staff members not cooperating with practice changes. This barrier may have been reflective of the environment in the care home, emphasising underlying problems that may have existed prior to trial commencement.

*“I would say in that home there’s two very definite groups of staff, the ones who want to see progress, who would support the mapper, who would want to encourage her and make it work, and there was also a very strong group of people who say you know ‘what she thinks she’s telling us’.” (DCM™ expert)*

As an intervention, DCM™ may be more accessible for larger care homes. This may be due to more qualified staff and greater access to funds and resources, for example with larger staffing pools to provide cover for mappers. Managers are an important influence in the delivery of DCM™, and can act as either a barrier or facilitator. A good relationship between the manager and mappers is crucial to successful implementation. Furthermore, having motivation and enthusiasm for making changes to practice was an important part of seeing benefit from DCM™ cycles. However, the challenges faced, such as staffing issues, sometimes overshadowed the motivation of individuals. Finally, as a care home level intervention, the engagement and motivation of staff was crucial to the successful implementation of DCM™. The mappers required leadership qualities and the respect of the staff team for DCM™ to have any influence in the care home. Without the support of the staff team, mappers struggled to make practice changes. These issues highlight the importance of managers selecting appropriate individuals to become mappers.

### Mapper barriers and facilitators

The selection of mappers had a significant impact on delivery of DCM™ as an intervention. During mapper recruitment, required qualities, including passion and enthusiasm to improve care and leadership skills to influence the staff team were considered. This was perceived as integral to DCM™ cycles taking place within the home.

#### Choice of mappers

Managers acknowledged that certain mapper qualities could facilitate DCM™ implementation. These included confidence in the DCM™ process, pragmatism, dedication, leadership abilities to engage staff and influence changes in practice, eagerness to learn and an overall interest in DCM™ and enthusiasm for improving dementia care. Managers were requested to identify mappers at the beginning of the trial, and they were subsequently recruited based on their relevant skills, but recruitment was also based on who was likely to maintain their role at the home for the trial duration. Whilst researchers provided the managers with guidance, the managers made the final decision.

*“Two team leaders stuck out a being really passionate about people living with dementia.” (Manager)*

Mappers having passion and motivation to improve the quality of care for people with dementia was found to be a facilitator in the effective implementation of DCM™. However, while initially motivated to engage in the mapper role, for one mapper, the challenges of implementing DCM™, particularly the lack of support, overruled her motivation and she became disengaged with the process. This indicates that even if mappers are selected for having appropriate skills and abilities, a failure in wider support can undermine this, demonstrating initial mapper passion or enthusiasm was not always enough to ensure successful DCM™ implementation.

*“It became a chore and one lady I can think of in particular was very excited and motivated about it, and became less so because of the challenges. And that’s really sad to see. Someone who had that real passion to just go “do you know it’s just too hard”, but initially is like “I’m happy to come in on my day off because I think it’s marvellous”, but when you’re not then getting that support it you know wears you out really. Wears you down.” (DCM™ expert)*

Practical skills and academic ability were also important to enable the chosen mappers to undertake the various tasks expected of them during in the DCM™ trial. These skills and abilities included computer literacy, writing high quality reports, fluency in English, and sufficient academic ability to undertake the more complex components. Mappers who did not possess the aforementioned capabilities, despite the trial processes used to identify and recruit appropriate mappers, could struggle to deliver the cycles of DCM™. Notably, poor IT skills and a lack of fluency in English were most frequently cited as barriers to successful DCM™ implementation.

Due to the identified skills and abilities of the individual, more senior members of staff were sometimes recruited as mappers, this had both positive and negative impacts on DCM™ implementation. Although senior staff were more likely to possess the necessary academic, writing and leadership skills to facilitate DCM™ implementation, it was often challenging to find protected time for staff in such roles to undertake mapping. Senior staff members who were recruited as mappers could be subject to multiple competing demands on their time, this challenging their ability to effectively deliver the DCM™ intervention.

*“[I chose] two quite strong team leaders that I knew would be able to get staff on their side and would be able to manage the feedback, because they can be quite difficult sometimes.” (Manager)*

Managers found the process of identifying and recruiting staff members who possess all or many of the necessary skills required to be a mapper difficult in the care home context.

*“If I look at the whole team there are few other people who would have been possible, academically capable of completing that project. And that’s a difficulty.” (Manager)*

An important factor in mapper selection was commitment, particularly in relation to the often distant geographical locations of training and the time commitment involved. These were logistical issues which were especially problematic for staff with caring or other commitments.

Management often had to prioritise availability over ability when making mapper choices, despite recognising the qualities that that were important for mappers to possess. In reality mapper choice often came down to who was available and willing to undertake the four-day course, particularly as this may involve travelling.

*“When we found out they would have to do four days training in London, [she] wasn’t able to do that. And because we found out almost at the last minute, we just had to grab somebody else that was free really”. (Manager)*

Again, following a mapper withdrawal, the manager chose an additional mapper based on their off-duty availability to attend the training course and not their abilities to facilitate implementation in the home.

*“I think when in one case where a manager … didn’t have a clue about who to nominate, she was just, she was looking at the off-duty and sort of picking names off the off-duty.” (DCM™ expert)*

One mapper reported feeling misinformed during the selection process, which may have impacted on their attitude towards DCM™.

*“We kind of got misled, I’ll be honest. It was something like a course came up and it were like if you want to learn more about dementia, which I did and [fellow mapper] did, and then we got, we just put us names forward.” (Mapper)*

#### Mappers experiences

Mappers who were less qualified or experienced reported greater difficulties in the DCM™ process, particularly in feedback and report writing, as they were asked to develop and utilise skills that they were not familiar with. Having the skills to facilitate and ask questions as part of feedback sessions that allowed staff members to give opinions rather than yes or no responses was particularly challenging for some mappers.

*“For some of the care workers writing anything was a real challenge. You know they just not, not used to putting descriptions down, let alone sort of feedback type questions to ask.” (DCM™ expert)*

Furthermore, there were many conflicting priorities placed on mappers. Some mappers felt they did not have protected time to implement DCM™ and were called upon to assist with direct care during mapping. This was particularly evident if the mapper had additional responsibilities, such as completing the medication rounds. This reduced the time available to implement DCM™.

*“I’d say it depends on your workloads and things that day, like how much is implemented. This thing took a lot of time, when there is not as many staff on as you need, and like I say, we have several people who are end of life, and things like that. Priorities are more that way at the time.” (Mapper)*

The interviewees suggest the selection of mappers had a significant impact on the success of DCM™ as an intervention. The selection of mappers with the aforementioned qualities facilitates the delivery of DCM™, as difficulties with stages of the process such as analysis, and report writing, can result in additional time being dedicated to the completion of cycles in an already time conscious setting. However, this was not always feasible. The completion of DCM™ cycles requires a level of commitment, both in effort and in time, some mappers had not anticipated or appreciated this when undertaking this role. Due to the amount of staffing time required to complete cycles, DCM™ was not viewed by some, in its current form, as a tenable intervention in a care home setting.

### Intervention barriers and facilitators

A number of barriers and facilitators related to the DCM™ intervention itself were identified and were perceived to have had an impact on how much implementation occurred within care homes. As the DCM™ process is complex, a clear understanding of the intervention was vital for DCM™ to be prioritised within the care home, despite its time consuming nature.

#### Understanding of DCM™

The extent to which mappers, managers and staff valued and understood the benefits of DCM™ influenced the success of its delivery. Care home staff were more likely to be engaged if they perceived DCM™ as a beneficial tool that could enhance care quality and improve quality of life for residents. However, challenges arose if care home staff did not clearly understand the DCM™ process, particularly regarding the required time commitments.

A clear understanding of the DCM™ intervention and its potential as a mechanism for changing the care delivered in care homes was crucial to the engagement of the home. Where some mappers did not fully understand the process following training, they struggled to promote the intervention to others within the home, leading to poor levels of engagement.

*“The trouble is, when they came back [from the training], they weren’t able to explain properly what they had to do. So, you know, they were trying to explain it to us and we were finding difficulty understanding what was actually involved.” (Staff Member)*

Where there were poor levels in mappers’ understanding of DCM™, a lack of engagement in the process filtered through the home. This caused DCM™ to be perceived as a distraction from direct care leading to incomplete intervention cycles.

*“I still don’t understand it … no one has been able to understand it to me fully… Every time I asked them [the mappers] to explain they were struggling. So I never got a full grasp of what it was all about.” (Manager)*

Essentially, for the majority of mappers, the benefits of utilising DCM™ as a tool for changing practice was apparent, even where this was not clear to their managers. This was particularly noted during the observation periods, where areas of good and poor care may have been observed.

*“You can see a big difference. You can actually see what goes on through their [the residents] eyes. When you sit there and watch them for about three hours.” (Mapper)*

These findings indicate the importance of mappers, managers and staff having a good understanding of the DCM™ intervention and the processes required before attempting to implement it. Without this understanding DCM™ is not seen as a priority within the care home.

#### Complex nature of DCM™

Some participants felt that the DCM™ intervention was overly complex and time consuming, which was felt to be a barrier to its implementation. Particular components of DCM™ were identified as being difficult, including the observation phase and associated coding, the report writing, and generally, the language used.

*“Some of the things that certainly I picked up on, some of the things they found more difficult was around the kind of data analysis and report writing. That was the area that people seemed to find most difficult.” (DCM™ expert)*

Certain aspects of the intervention were identified as time consuming or overly onerous, including the length of the training course, and the paperwork and report writing requirements.

*“So the report writing, yeah, was horrific to be honest. Very time consuming. Obviously we both had different roles at that point so quite demanding, so getting time, and it’s not a very quick process. Like I say it took quite a lengthy period of time … it was very demanding.” (Mapper)*

Some mappers reported that delays between them completing the training course and initiating their first cycle of DCM™ led to them forgetting some of the finer details, such as the observational coding framework. To help with the issue, DCM™ experts were able to provide mappers with additional time and support to ‘revise’ some parts of their training before starting the mapping cycle.

The length of time required to undertake DCM™ meant that mappers had to be taken away from their usual roles and defined as ‘off the floor’, therefore removed from the core business of care delivery in the case of direct care staff. This posed a strain on both the care home resources and the staff members involved. Ahead of participation, management agreed to provide additional time for mappers, however it was frequently reported that this had not been provided, with many mappers completing tasks outside of working hours.

*“The mappers were also carers and nurses and had, you know, activities and tasks and jobs to do as well as the mapping. Yeah, I think they found it quite overwhelming.” (Manager)*

In addition, some managers felt that once the training course was completed they were then left to implement DCM™ on their own, although in reality every home had access to a DCM™ expert for 5 days to support implementation of their first cycle. Of the 31 homes who received the intervention, only two did not utilise the expert support at all, and four did not utilise all 5 days. Such views raise questions about the fit of DCM™ for care homes when led by care staff and suggest the need to consider adapting standard DCM™ processes for care home staff in the future development of the tool.

### Trial barriers and facilitators

Participant expectations held before participating in the trial did not match the realities of implementing DCM™. The combined burden of trial expectations and DCM™ participation proved difficult for some care homes to manage.

#### Expectations of the trial

Most generally, the time required to complete cycles of DCM™ far exceeded the expectations of the managers and mappers. This impacted on the schedules in place for each care home and led to expert mappers consistently renegotiating schedules.

*“I had to do my normal working hours, plus a lot of the time, a lot of extra hours, because we were short. I was sometimes doing 40 odd hours a week. Then, coming in and trying to do the typing up on top of that, especially the last one with it being over Christmas - it was taking a lot longer to do, than I would have liked really.” (Mapper)*

Some managers reported being unaware that the mappers could not be included as members in the staff team and thus could not provide direct care on the days they were mapping. These managers did not appreciate that the mappers were unable to stop mapping to assist residents with any care needs they had during the observation process. This led to tensions between some managers, mappers and expert mappers.

*“…they didn’t realise that they would have to be off the numbers to do the you know, preparing the map, for the mapping, for the map itself and to do the rest of their work.” (DCM™ expert)*

The care homes were provided with detailed written and verbal explanations of the trial process, expectations and time commitments from the research team before agreeing to participate. Yet it is clear from the data that the management and staff expectations of the trial differed from the realities of participation.

#### Input from DCM™ expert mappers

DCM™ expert mappers viewed themselves as incredibly valuable to the implementation of DCM™, suggesting that without their input and support, DCM™ would not have not been successfully implemented in many care homes.

*“If the expectations had remained the same, I don’t think it would have worked without the expert mappers.” (DCM™ expert)*

The expert mappers provided valued support during the first cycle of DCM™, helping to clarify any uncertainties and alleviate mapper doubts.

*“It is nice to have somebody sat with you whilst you’re actually doing it practically, to be able to say ‘Am I using this code or that code?’ ‘Am I observing this right?’” (Mapper)*

When DCM™ expert mapper support was delivered flexibly, in a friendly manner, it was valued by care homes. There were, however, times when this support was problematic and not well received.

*“The expert mapper was a little full on. Knew her subject, very passionate, but very, erm, timescale orientated. Which… I think, added to the stress.” (Manager)*

The DCM™ expert mappers perceived that they went above on beyond their expected roles to provide support. Whilst five days were allocated to support each care home, certain situations led to increased need for DCM™ expert mapper support, such as a care home having only one mapper, or tensions between mappers.

*“I’ve tried to support her individually because the other mapper hasn’t supported her in the individual care summaries. So I’ve tried to support her extra by phone and do that, but I don’t think … she had the skills to do that by herself.” (DCM™ expert)*

The management of some homes did not feel supported, despite support from the DCM™ expert mapper being provided to all homes during the first cycle of DCM™.

*“I feel as if we were, had the training and then left to our own devices really.” (Manager)*

Conversely, some mappers felt that they did not require the support and that as they know the residents well, they had a better insight into the residents than the DCM™ expert.

*“When you learn anything really you just want to go and do it on your own don’t you. You don’t want someone looking over your shoulder going: yeah, yeah you’ve not done that right, or I didn’t get that or why did you put that … well I know that resident and I know.” (Mapper)*

For other mappers, the DCM™ expert mappers input was valued during the first cycle but it was felt that they required more support than was provided, to continue to undertake DCM™ cycles and would have benefitted from expert mapper support throughout all three cycles.

*“When she’d gone the support had gone” (Mapper)*

It was suggested by a DCM™ expert mapper that in the future, research assistants should offer their support to mappers to complete the required paperwork. However, this is not representative of the typical use of DCM™ within care homes, which was critical to the current trial examining DCM™ in a pragmatic fashion.

*“I think you would’ve really struggled if they hadn’t had someone going in. Be that an expert mapper or be that a research assistant, to go in and support them with doing the paperwork and completing that … they would need some kind of support to be able to engage with the research.” (DCM™ expert)*

In summary, DCM™ expert mappers felt that they had a positive effect on DCM™ implementation and as a result, felt that substantially more DCM™ cycles were completed. However, this view was not always shared by mappers and managers. The implementation data, which showed only 26% of intervention homes completed further, acceptable DCM™ cycles after the expert supported first cycle (see Table 3), suggests the value of expert mapper input for supporting DCM™ implementation in care home settings.

## Discussion

There were many barriers and facilitators to implementing DCM™, due to the complex nature of both the intervention and care home settings. These operated at a care home, intervention, and mapper level, with additional trial participation related barriers identified.

The culture of the care home was particularly important, ensuring that good relationships existed between mappers, managers and the wider staff team. This echoes the findings of Quasdorf and colleagues [11] who found that in order for DCM™ to be successfully implemented, the organisational context must include staff teams with little turnover, who communicated well and without hierarchies. However, this is an idealistic view and it is not clear how realistic such a structure is. Furthermore, lack of exposure to a person-centred approach is a barrier to DCM™ implementation [1]. This suggests that prior to implementation of DCM™, care home teams should be encouraged to evaluate their culture and identify whether any preliminary work needs to be done to allow DCM™ to be successfully implemented into the care home. This may include team building exercises [11] and identifying gaps in staff knowledge, to encourage a culture with a person-centred focus. As DCM™ needs to be integrated into practice over a period of time into order to be effective [15], it may also be important for DCM™ to be seen as a component of care homes’ long term plans, rather than a standalone intervention. This should include an organisational commitment to delivery of person-centred care and a culture that is supportive of such practices [12].

Selection of appropriate staff as mappers was key, ensuring that they had skills to implement DCM™, including suitable written and verbal language skills, the time to undertake all aspects of DCM™ within their day to day role, being well respected by the staff team, and having leadership capabilities and influence among staff. It was crucial that the expectations and value of DCM™ were understood by both managers and mappers before training was completed. These findings support earlier process evaluations, which highlighted the importance of positive attitudes towards DCM, particularly amongst managers and team leaders [1, 11]. Specifically, leadership within a care home needs to include and empower all staff in the process for DCM™ to be successfully implemented [6].

These findings also have implications for the DCM™ tool itself, with staff reporting feeling a lack of confidence in implementing it as well as a lack of time to complete the full process. This was despite undertaking 4 days of training, which was felt to be onerous by staff but also inadequate to prepare them to undertake DCM™. All components of the process were felt to be too complex, with expert mappers identifying that report writing and action planning were particularly difficult for staff to complete. Given the known contextual challenges faced by care homes including low staff literacy [16], numeracy, IT skills and accessibility and lack of time and resources [17], the complexity of DCM™ warrants consideration. If DCM™ is to be used in the future within care home settings, by care home staff, consideration of the ways in which the process could be shortened or simplified may be beneficial to supporting implementation.

Implementation was easier in larger care homes, where there was budget to allow mappers to be released from their usual roles. The support of expert mappers was particularly important in the beginning to implement DCM™. This is not a standard component of DCM™ unless purchased as an addition to training. To our knowledge, this trial was the first to include such a support system, but the findings suggest that it was felt to be necessary by the majority of mappers during the trial. This finding echoes the observations within a recent systematic review [12] and has implications for considering the way that mappers are currently trained and the support that may be required for them to fully engage in the 4 phases of a DCM™ practice development cycle. This is supported by other research findings, which suggest that implementation of new psychosocial interventions require a period of sustained supervision (e.g. [18]).

Overall, the evidence suggests a range of factors that may influence whether care homes are able to successfully implement DCM. This paper has not explored the combinations of these factors and their potential association with greater or poorer implementation. This is something that should be explored further in order to identify whether there appear to be crucial combinations of factors that lead to success or not.

There are several limitations with the present study. Firstly, interviews were only conducted in 18 of the 31 intervention care homes. Those not involved in the Process Evaluation may have had additional insights to offer. Some residents and relatives were asked for their views on DCM™ implementation but were unable to comment specifically on this. To date, no studies of DCM™ implementation have interviewed residents or relatives, therefore we were unsure how well they would be able to contribute to our understanding of intervention implementation. Our findings suggest that many residents and relatives remain largely unaware of ongoing interventions in care homes even when participating in a trial of the intervention. However, their awareness may also have been affected by poor implementation and/or a failure in some care homes to involve residents or relatives in the DCM process. As the involvement of relatives and residents is key in practice change in long term care (e.g. [19]), this may account for some of the lack of success. In addition, residents with dementia experienced difficulties in recalling past events and experiences of interest during interviews. Future care home trials incorporating a process evaluation should consider using researchers independent of the trial to enable concurrent collection of trial and process data during intervention implementation; this may increase opportunities for residents and relatives to comment on the intervention and its effects as they occur. Furthermore, individuals who felt strongly about DCM™, either positively or negatively, may have been more likely to agree to participate in an interview, providing a sample that may not fully reflect experiences from the trial. Additionally, some Managers, mappers and other staff who were present at the outset and earlier stages of the trial were no longer in post during the process evaluation data collection and therefore their experiences were not captured. The interviews within the present work were conducted after implementation had been completed, meaning that it may have been difficult for participants to accurately recall details of the intervention, particularly the earlier stages. However, as we were expecting DCM™ to be implemented as it would in the real-world setting, conducting interviews during the process would have been likely to bias implementation. As researchers were blinded to intervention allocation, it would also have required considerable additional trial resource.

Considering the results of the present work alongside the findings from previous explanatory trials of DCM™ indicates that externally led or supported implementation of DCM™ may provide a more beneficial and sustainable format for DCM™ delivery (i.e. [5, 6]). This aligns with the broader contextual challenges faced by care homes in implementing complex interventions that are staff led, these include but are not limited to high staff turnover rates; low staff educational levels [20] and confidence; and lack of time and resources. Future research will need to consider mechanisms for addressing these wider contextual issues within the context of intervention design and delivery. Utilising ‘bottom up’ approaches to intervention design, that involve care home staff, managers and providers may provide a mechanism to identify and address potential challenges within the development process.

This study indicates that the involvement of care home management from the beginning of the process, ensuring that they have a full understanding of the commitments of implementing DCM™ and the support that their mappers will require, before staff members attend training, is vital for successful DCM™ implementation. Selecting the right people to undertake the mapper role is also crucial and in choosing individuals, managers should look not only at the qualifications they hold, but also their confidence, IT, and leadership skills, ability to engage and motivate staff members, and to positively challenge current poor practice within the care home.

There were issues with implementation of DCM™ in most of the care homes within this study, raising questions around the appropriateness of DCM™ in its current form for care homes. In order for DCM™ to continue to be used in care homes, those responsible for training individuals in its use need to reconsider the amount of time needed and paperwork that mappers are asked to complete, as well as the complexity of using the tool.

This study suggests a range of recommendations for practice that researchers and care homes should consider before implementing DCM™. These may also be helpful to consider when implementing other similar, complex psychosocial interventions. For example, it is important for researchers to take time to understand the culture of the care home to ensure it is one that will be supportive and open to use of DCM™. This may include assessing organisational, manager and staff openness to practice change and their commitment to delivering person-centred care. Where settings are not yet ready for DCM™ additional work may need to be undertaken, potentially over a sustained period, in order to develop the required supportive context. This might include additional training for staff and work with the management team on openness to and support for change. Ensuring the care home manager is fully committed to the process and is willing to engage with and support its implementation on a sustained basis is also crucial. The manager needs to have a thorough understanding of DCM™ and how it is implemented, in order to do this. Consideration of alternative models of DCM™ may also be advantageous, given the high staff turnover rates in care homes and the frequent difficulties we found that care home managers had in identifying care home staff who had the full range of skills and qualities needed to successfully train in and then implement DCM™. This study has demonstrated that training two members of care home staff is unlikely to be practical or sustainable model of DCM™ implementation for most care homes. Where a care provider has a number of care homes, this might for example, include development of a central team of skilled staff who undertake DCM™ across a number of homes and who work with their staff to support implementation of action plans and practice change. These recommendations also indicate that it may be helpful for an individual who is skilled in the implementation of DCM™ to support care homes to assess their readiness for DCM™ ahead of them making a decision to train staff and implement the method.

## Conclusions

Barriers and facilitators to DCM™ implementation were found at the mapper level, the DCM™ intervention level and the care home level. Further barriers caused by the burdens of trial participation were also identified. Ensuring that the intervention is well understood by staff teams and that the expectations of the management are realistic may be key to successfully implementing DCM™. As better implementation of DCM™ is thought to improve its effectiveness [5], understanding the barriers and facilitators to implementation and sharing best practice models of implementation with practitioners and research teams is crucial to support future use of DCM™. This may include exploring alternative models of implementation that do not rely on care home staff members to undertake the mapper role, particularly if no staff members can be identified who possess the required skills and qualities to lead DCM™ implementation.

## Abbreviation

DCM™: Dementia Care Mapping
